# Oral transfer of chemical cues, growth proteins and hormones in social insects

**DOI:** 10.7554/eLife.51082

**Published:** 2019-08-19

**Authors:** Adria C LeBoeuf, Patrice Waridel, Colin S Brent, Andre N Gonçalves, Laure Menin, Daniel Ortiz, Oksana Riba-Grognuz, Akiko Koto, Zamira G Soares, Eyal Privman, Eric A Miska, Richard Benton, Laurent Keller

LeBoeuf AC, Waridel P, Brent CS, Gonçalves AN, Menin L, Ortiz D, Riba-Grognuz O, Koto A, Soares ZG, Privman E, Miska EA, Benton R, Keller L. 2016. Oral transfer of chemical cues, growth proteins and hormones in social insects. *eLife* **5**:e20375 . doi: 10.7554/eLife.20375.

Published 29, November 2016

Prof. Jocelyn Millar has pointed out some errors in our GC-MS-based identifications of hydrocarbons detected in trophallactic fluid (TF) (Table 1). With his feedback, we have refined our method of assignment to account for ambiguity due to the sparse representation of these compounds in the NIST Webbook, and reanalyzed the data.

We have made the necessary corrections to Table 1, the paragraph on these results the main text, the legend for Figure 3, Figure 3 - figure supplement 1 and the "Identification of trophallactic fluid hydrocarbons" section of the methods. We regret the presence of these errors in our published manuscript but would like to stress that the fundamental conclusions of the paper remain unchanged. We thank Prof. Millar for pointing out this issue.

## Table 1

The corrected Table 1 is shown here:

**Table:** 

Rt (min)	MW	Proposed MF	Proposed structure	RI(a)	Peak ID
13.35	212	C15H32	Pentadecane	1500	
14.05	226	C16H34	5-methylpentadecane	1541	
15.05	226	C16H34	Hexadecane	1600	
16.47	240	C17H36	Heptadecane	1700	
17.06	254	C18H38	7-methylheptadecane	1742	
17.83	254	C18H38	Octadecane	1800	
19.15	268	C19H40	Nonadecane	1900	
19.89	295	C21H44	*-trimethyloctadecane	1950	
19.97	256	C16H32O2	n-Hexadecanoic acid	1956	
20.17	282	C18H34O2	Hexadecenoic acid, ethyl ester	1970	
20.50	284	C18H36O2	Ethyl palmitate	1992	
20.62	282	C20H42	Eicosane	2000	
20.98	268	C18H36O	Octadecanal	2020	
22.89	280	C18H32O2	Linoleic acid	2125	
23.03	282	C18H34O2	Oleic acid	2137	●
23.46	308	C20H36O2	*-Octadecadienoic acid, ethyl ester (possibly Ethyl linoleate)	2155	
23.59	310	C20H38O2	Ethyl oleate	2165	●
24.30	310	C22H46	Docosane	2200	
26.27	322	C23H46	*-tricosene	2279	●
26.56	324	C23H48	Tricosane	2300	
29.08	338	C24H50	Tetracosane	2400	
30.76	352	C25H52	2-methyltetracosane	2461	
31.05	350	C25H50	*-pentacosene	2472	
31.26	350	C25H50	*-pentacosene	2480	
31.80	352	C25H52	Pentacosane	2500	
34.69	366	C26H54	Hexacosane	2600	
36.37	380	C27H56	4-methylhexacosane	2658	
36.91	378	C27H54	*-heptacosene	2675	
37.70	380	C27H56	Heptacosane	2700	
39.19	394	C28H58	5-methylheptacosane	2755	
40.63	394	C28H58	Octacosane	2800	
41.36	422	C30H62	*-trimethylheptacosane	2835	
41.88	408	C29H60	4-methyloctacosane	2860	
42.23	406	C29H58	*-nonacosene	2877	
42.47	422	C30H62	2,10-dimethyloctacosane	2889	G
42.70	408	C29H60	Nonacosane	2900	H
42.90	422	C30H62	*-dimethyloctacosane	2918	
43.29	422	C30H62	9-methylnonacosane	2938	
43.36	422	C30H62	7-methylnonacosane	2942	
43.51	422	C30H62	5-methylnonacosane	2951	
43.79	436	C31H64	7,11-dimethylnonacosane	2968	
43.90	422	C30H62	3-methylnonacosane	2976	E
43.96	436	C31H64	5,9-dimethylnonacosane	2980	K
44.12	450	C32H66	*-trimethylnonacosane	2991	
44.42	436	C31H64	3,7-dimethylnonacosane	3008	B
44.74	450	C32H66	3,7,11-trimethylnonacosane	3036	Q
45.01	464	C33H68	3,7,11,15-tetramethylnonacosane	3056	
45.05	436	C31H64	*-methyltriacontane (likely 4-)	3060	R
45.30	434	C31H62	*-hentriacontene	3074	
45.45	450	C32H66	4,10-dimethyltriacontane	3088	S
45.54	386	C27H46O	Cholesterol-like	3090	A
45.70	436	C31H64	Hentriacontane	3100	M
46.00	464	C33H68	*-dimethylhentriacontane (likely 9,13)	3139	C
46.56	464	C33H68	5,9-dimethylhentriacontane	3187	D
46.69	492	C35H72	*-pentamethyltriacontane (possibly 7,11,15,19,23)	3199	J
46.93	478	C34H70	*-trimethylhentriacontane (likely 3,7,11-)	3223	O
47.15	492	C35H72	5,9,11,15-tetramethylhentriacontane	3245	N
47.59	492	C35H72	*-trimethyldotriacontane or *-tetramethylhentriacontane	3289	P
48.78	506	C36H74	*-Trimethyltritriacontane (possibly 9,13,17)	3412	L
49.67	520	C37H76	*-trimethyltetratriacontane	3500	
50.28	534	C38H78	*-tetramethyltetratriacontane	3554	

The original Table 1 is shown here:

**Table:** 

Retention Time	Proposed MF	Proposed structure	RI	Peak ID
13.35	C_15_H_32_	Pentadecane	1500	
14.05	C_16_H_34_	4-Methyltetradecane	1600	
14.34	C_10_H_10_O_3_	Mellein	1674	
15.05	C_16_H_34_	Hexadecane	1600	
16.47	C_17_H_36_	Heptadecane	1700	
17.06	C_20_H_42_	7,9-Dimethylheptadecane	1710	
17.83	C_18_H_38_	Octadecane	1800	
19.15	C_19_H_40_	Nonadecane	1900	
19.89	C_21_H_44_	7,10,11-Trimethyloctadecane	1920	
19.97	C_16_H_32_O_2_	n-Hexadecanoic acid	1962	
20.5	C_18_H_36_O_2_	Ethyl palmitate	1968	
20.62	C_20_H_42_	Eicosane	2000	
20.98	C_18_H_36_O	Octadecanal	1999	
22.89	C_18_H_32_O_2_	Linoleic acid	2133	
23.03	C_18_H_34_O_2_	Oleic acid	2179	•
23.46	C_20_H_36_O_2_	Ethyl-9-Cis-11-Trans-octadecadienoate	2193	
23.59	C_20_H_38_O_2_	Ethyl oleate	2173	•
24.3	C_22_H_46_	Docosane	2200	
26.27	C_23_H_46_	7Z-Tricosene	2296	•
26.45	C_20_H_38_O_2_	Cis-13-Eicosenoic acid	2368	
29.08	C_24_H_50_	Tetracosane	2400	
32.51	C_25_H_52_	Pentacosane	2500	
34.69	C_26_H_54_	Hexacosane	2600	
36.37	C_27_H_56_	4-Methylhexacosane	2640	
36.91	C_27_H_54_	Heptacosene	2672	
38.76	C_28_H_58_	9-Methylheptacosane	2740	
39.19	C_28_H_58_	5-Methylheptacosane	2740	
40.05	C_28_H_58_	*-Trimethylpentacosane	2610	
40.63	C_28_H_58_	Octacosane	2800	
40.76	C_28_H_58_	7-Methylheptacosane	2753	
41.36	C_29_H_60_	5,7,11-Trimethylhexacosane	2783	
41.88	C_29_H_60_	4-Methylnonacosane	2810	
42.23	C_29_H_58_	Nonacosene	2875	
42.47	C_30_H_62_	2,10-Dimethyloctacosane	2874	G
42.7	C_29_H_60_	Nonacosane	2900	H
42.9	C_31_H_64_	9,16-Dimethylnonacosane	2974	
43.29	C_31_H_64_	9,20-Dimethylnonacosane	2974	
43.36	C_30_H_62_	7-Methylnonacosane	2940	
43.51	C_31_H_64_	4-Methyltriacontane	3045	
43.79	C_31_H_64_	7,16-Dimethylnonacosane	2974	
43.9	C_31_H_64_	2-Methyltriacontane	3045	E
43.96	C_31_H_64_	10-Methyltriacontane	3045	K
44.12	C_32_H_66_	10,11,15-Trimethylnonacosane	3023	
44.42	C_32_H_66_	*-Dimethyltriacontane	3083	B
44.74	C_32_H_66_	8,12-Dimethyltriacontane	3083	Q
45.02	C_33_H_68_	*-Trimethyltriacontane	3119	R
45.3	C_31_H_62_	Hentriacontene	3100	
45.45	C_33_H_68_	5,10,19-Trimethyltriacontane	3119	S
45.54	C_27_H_46_O	Cholest-5-en-3-ol / Cholesterol	3100	A
45.7	C_31_H_64_	Hentriacontane	3100	M
46	C_34_H_70_	9,13-Dimethyldotriacontane	3185	C
46.56	C_33_H_68_	5,9-Dimethyldotriacontane	3185	D
46.69	C_34_H_70_	*-Tetramethylnonacosane	3160	J
46.93	C_34_H_70_	*-Multiramified tetratriacontane	3220-3100	O
47.15	C_34_H_70_	5,9,13,17,21-Pentamethylnonacosane	3100	N
47.28	C_33_H_68_	*-Dimethylhentriacontane	3185	T
47.58	C_34_H_70_	10,14,18,22-Tetramethyldotriacontane	3160	P
48.01	C_34_H_70_	*-Tetramethyldotriacontane	3160	
48.12	C_35_H_72_	11,15-Dimethyltritiacontane	3380	F
48.77	C_35_H_72_	*-Methyltetratriacontane	3440	L
49.54	C_36_H_74_	14-Methylpentatriacontane	3540	
49.67	C_36_H_74_	*-Multiramifiedhexatriacontane	3420-3300	
50.28	C_37_H_76_	*-Tetramethyltetratriacontane	3480	

## Main results

The corrected section of the main results:

“We identified 61 molecules in TF (Table 1), including fatty acids and fatty acid esters, linear alkanes, double bonded hydrocarbons, branched hydrocarbons, and a cholesterol-like molecule. The most abundant TF compounds comprised 27 or more carbons.”

These changes were made to reflect the updated identifications and improve the clarity that these are the molecules we were able to identify rather than a complete list of all components found in TF. The last sentence places the emphasis on the molecules of interest (i.e., those abundant in TF) rather than on the molecules we were able to identify.

The original section of the main results:

“We identified 63 molecules in TF (Table 1), including eight fatty acids and fatty acid esters, 13 unbranched and 36 branched hydrocarbons with one to five methyl branches. The majority (40/63) of the TF compounds comprised 27 or more carbons.”

## Figure 3 legend

The corrected legend for Figure 3 is shown here:

… "The abundant component (peak A) found in TF samples but not on the cuticle was a cholesterol-like molecule that insects cannot synthesize but must receive from their diet. Three molecules outside this window were found only in TF and not on the cuticle: *-tricosene, oleic acid, ethyl oleate (Table 1)”….

The original legend for Figure 3 is shown here:

… “The abundant component (peak A) found in TF samples but not on the cuticle was cholesterol, a molecule that insects cannot synthesize but must receive from their diet. Three molecules outside this window were found only in TF and not on the cuticle: 7Z-tricosene, oleic acid, ethyl oleate (Table 1).”…

## Methods

A number of changes were made to the section of the Methods, “Identification of trophallactic fluid hydrocarbons”. These changes were made to reflect the updated method for identification, the updated identifications and to improve clarity. This section of the methods is shown here in full with corrected sections in italics.

“Characterization of branched alkanes by GC-MS remains a challenge due to the similarity of their electron impact (EI) mass spectra and the paucity of corresponding spectra listed in EI mass spectra databases. A typical GC-MS chromatogram (Figure 3—figure supplement 1A) reveals the complexity of the TF sample. The workflow described here was systematically used to characterize the linear and branched hydrocarbons present in TF samples summarized in Table 1. The parent ion was first determined for each peak after background subtraction. Ambiguities remained in some cases due to the low intensity or absence of the molecular ion.

Linear alkanes present in TF samples were localized using a standard mixture of C8-C40 alkanes. Then RI values were deduced for all compounds present in the samples based on their retention times (Figure 3-figure supplement 1G, red).

*To determine the number of methyl branches for alkanes, we examined the distribution of fragment ions in the spectrum by fitting their intensities with an exponential decay function and specifically looking for the fragment ions that do not fit to the calculated exponential decay function*. From the experimental mass spectrum (Figure 3—figure supplement 1B–C), the intensity of all fragment ions was extracted and fitted *with the function *(Figure 3—figure supplement 1D–E). Figure 3—figure supplement 1 clearly shows two different resulting EI mass spectra profiles: on the left a linear alkane corresponding to *n-*hexacosane (Rt = 34.69 min, MF C_26_H_54_); on the right, *a monomethyl-branched* structure most likely corresponding to *9-methylnonacosane* (Rt = 43.29 min, MF *C_30_H_62_*) with two enhanced fragment ions *at m/z 141 and 309 emerging from the curve. *

Extracting and fitting the fragment ion profiles first helped to discriminate between linear and branched hydrocarbons, but also to estimate the number of methyl branches. To confirm for each compound the number of carbons and branches obtained, we used Kovats retention index (RI) values in the NIST Chemistry WebBook (Linstrom et al., 2000). Based on the RI values for similar GC stationary phase from C15 to C38 hydrocarbons, six different curves of RI vs. number of carbons were constructed from linear to pentamethylated alkanes (Figure 3—figure supplement 1F). To construct the curves, average RI values were taken of all hydrocarbons available in the database, with a given number of carbons and a given number of methyl branches. For example, the RI value of 2409 obtained for C_25_ and two branches is the average of 3,(7/9/11/13)-dimethyltricosane, 3,(3/5)-dimethyltricosane and 5,(9/11)-dimethyltricosane values listed in the NIST Chemistry WebBook for the same stationary phase. Those curves were used to check for every compound that, from the measured RI value and the number of carbons found, the number of ramifications found fit properly with the curves.

Once the number of branches and the parent ion mass were known, the position of the different branches could be deduced *from the fragment ion values. When ambiguity remained on the position of the branch or double bond, this is indicated with an asterisk in Table 1.*

The plot of experimental retention times for each compound as a function of the RI index closely fitted the plot using RI values given by NIST for the identified compounds (Figure 3—figure supplement 1G), bringing additional confidence to the identifications.

*Table 1 summarizes the* proposed structures for hydrocarbons and other compounds detected in TF samples.”

## Figure 3—figure supplement 1

The corrected Figure 3—figure supplement 1 is shown here:

**Figure 3—figure supplement 1.**
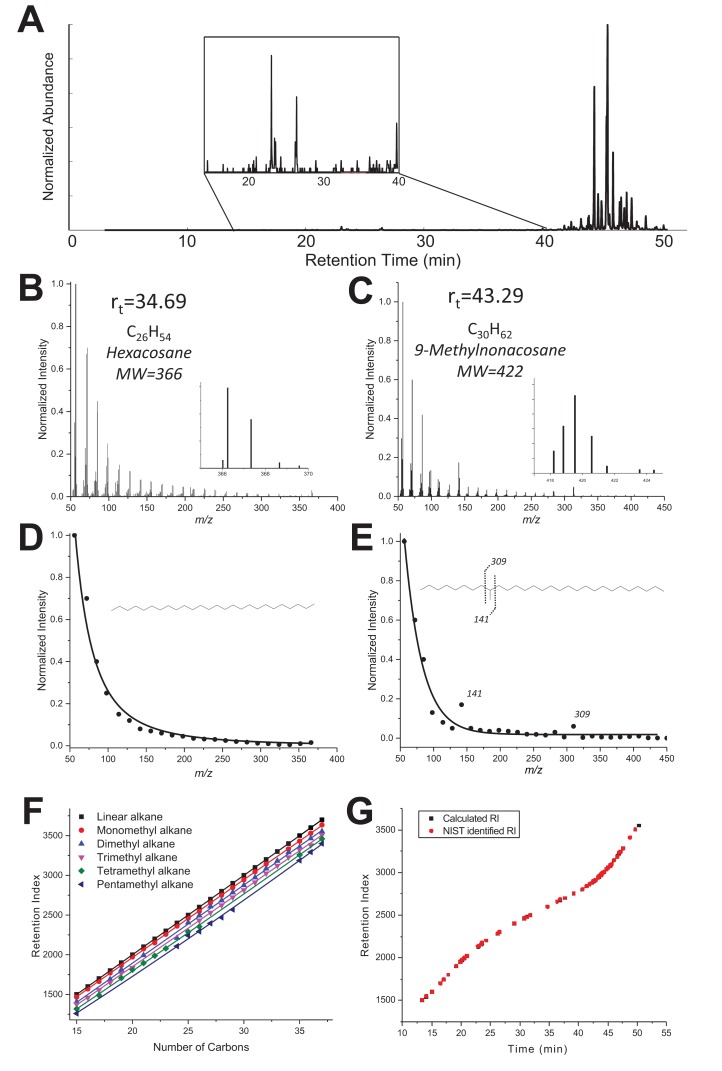


The original Figure 3—figure supplement 1 is shown here:

**Figure 3—figure supplement 1.**
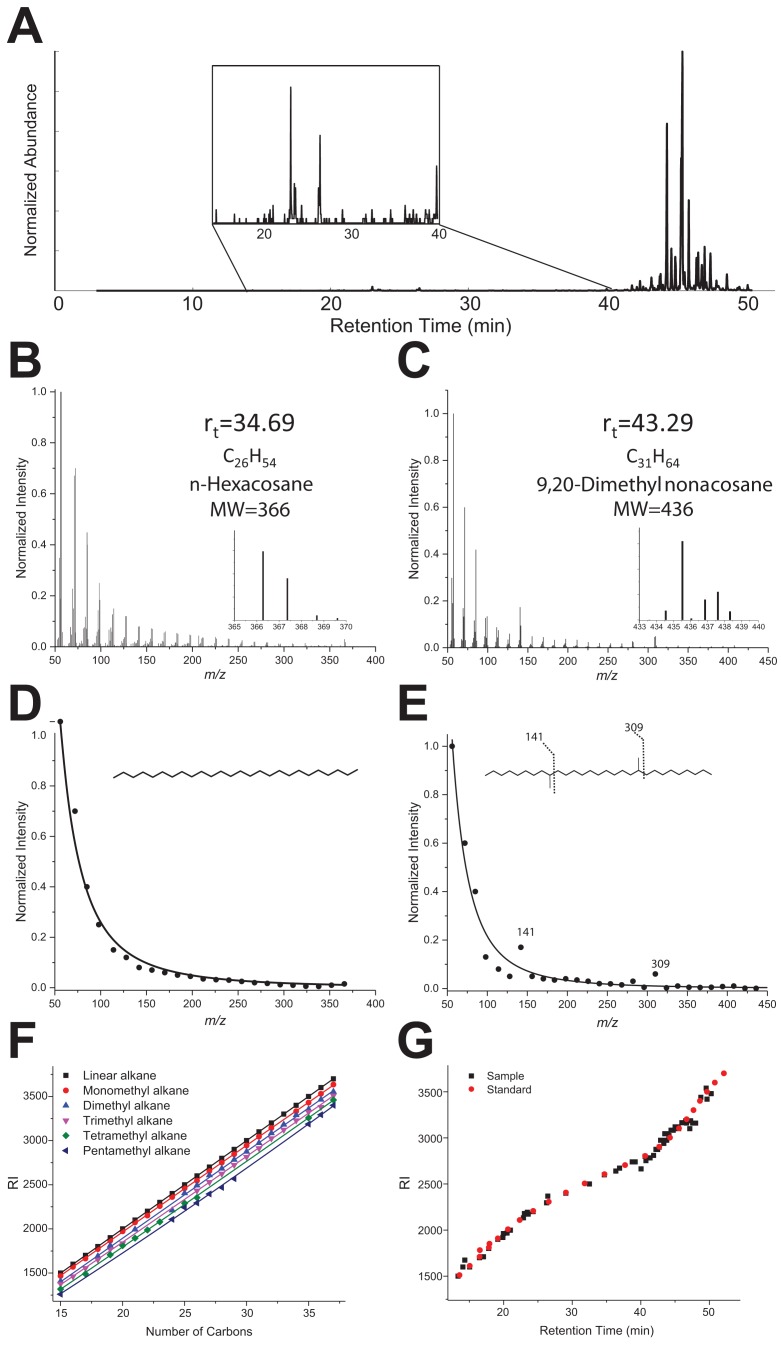


## Legend for Figure 3—figure supplement 1

Changes were made to reflect the updated identifications, to correct typographical errors, and to improve clarity of the methods.

Corrected legend (corrections shown in italics):

… “(**B–C**) GC-MS Mass spectra of a linear alkane *(C26, elution time 34.69 min, panel B) and a methylated alkane (C30, Rt 43.29 min, panel C). (**D-E**)* Extracted MS spectra were fitted with an exponential decay equation *with their proposed structure based on enhanced fragment ions (m/z 141 and 309 in the case of 9-methylnonacosane). (**F**)* RI values vs. number of carbons extracted from the NIST Chemistry WebBook library, depending on the number of methyl branches: linear (black), monomethyl (red), dimethyl (blue), trimethyl (pink), tetramethyl (green) and pentamethyl (dark blue) *alkanes. (**G**) Retention index values calculated for each compound listed in Table 1 are based on the elution time and RIs of a hydrocarbon ladder from C8-C40 (black) overlaid with NIST RI values reported in the NIST database for the identified hydrocarbons* (red).”

Original legend:

… “(**B–D**) GC-MS Mass spectra of linear alkane (*n*-hexacosane at Rt 34.69 min, panel B) and a dimethylated alkane at Rt 43.29 min (**C**). Extracted MS profiles were fitted with an exponential decay equation (**B, D**). The proposed structure for the branched alkane based on fragments ions (141 and 309) is 9,20-dimethyl nonacosane (**D). (E**) RI values vs. number of carbons extracted from the NIST Chemistry WebBook library, depending on the number of ramifications: linear (black), monomethyl (red), dimethyl (blue), trimethyl (pink), tetramethyl (green) and pentamethyl (dark blue). (**F**) The black dots represent experimental retention times with the calculated RI index for all identified hydrocarbons of the TF sample. The red dots represent the experimental retention times and with their RI index for the linear C8-C40 alkane standard.”

The article has been corrected accordingly.

